# New Frontiers in Computer-Assisted Career Guidance Systems (CACGS): Implications From Career Construction Theory

**DOI:** 10.3389/fpsyg.2022.786232

**Published:** 2022-02-15

**Authors:** S. Alvin Leung

**Affiliations:** Department of Educational Psychology, Faculty of Education, The Chinese University of Hong Kong, Hong Kong SAR, China

**Keywords:** computer-assisted career guidance system, career assessment, career construction, career guidance, career intervention, life design career counseling

## Abstract

This article addresses the use of computer-assisted career guidance systems (CACGS) in career interventions. Major CACGS developed in the past decades were based on the trait-factor or person-environment fit approaches in their conceptualization and design. The strengths and limitations of these CACGS in addressing the career development needs of individuals are discussed. The Career Construction Theory (CCT) is a promising paradigm to guide the development of new generations of CACGS. The narrative tradition, career adaptability model, and life-design interventions of CCT offer rich conceptual and practical applications that could expand the scope and breadth of career exploration and identity construction through using CACGS. A digital system developed in Hong Kong called Infinity is a case-in-point of a CACGS where users could learn about career planning, engage in self and career construction through using the quantitative and qualitative assessment applications and gamified tools, take career planning actions over time, and communicate with their social supportive systems. Initial findings suggested that users of the system reported lesser decision-making difficulties, higher levels of decision clarity, and better understanding of what is career planning than non-users. Users from high academic achievement schools reported higher levels of career adaptability than their counterparts in schools of similar academic background. Users from low achievement schools reported higher intention to pursue government-supported universities than non-users from schools of similar background. Research and practice implications in schools and organizational settings are discussed.

## Introduction

Digital career development and planning systems are useful and convenient modes of career intervention in a world that is increasingly fast-changing and unpredictable. In-person career counseling and interventions are irreplaceable but a good digital system can offer alternative means for individuals to learn about career planning and cope with career transitions (e.g., Betz and Borgen, 2010; Copeland et al., 2011; Vigurs et al., 2017). Sampson and Osborn (2015) identified multiple benefits from using information and communication technology (ICT) in career guidance, including (a) increased access to information, (b) improved access to service for individuals in remote areas or with special barriers, (c) rapid information made available by search engines, (d) use of interactive and multimedia materials to maximize learning, and, (e) cost effectiveness.

Sampson and Osborn (2015) identified three categories of ICT applications in career development, which are computer-assisted career assessment, computer-assisted career information, and computer-assisted career guidance systems. Computer-assisted career assessment systems are adaptations of paper-and-pencil career development instruments in a digital platform (e.g., web-based). Users receive a test report from the system soon after completing the assessment. Digital technology allows for a high degree of accuracy in scoring, personalized test results interpretation, and visually-enhanced profiles. Computer-assisted career information systems are platforms of occupational, educational, employment, and job-related information. Information is presented in different media formats, including but not limited to written narratives, videos, and simulations (e.g., virtual-reality). Meanwhile, computer-assisted career guidance systems (CACGS) are those that seek to guide users through a process of career assessment and career information exploration. A distinguishing characteristic of CACGS is that the steps of assessment and career information exploration are connected. Inputs and outcomes from one of the steps (e.g., assessment) could inform and interact with the other steps of the system (e.g., information exploration) to help users achieve career development objectives (e.g., informed career and educational choices).

The classification by Sampson and Osborn (2015) could not account for the fast-changing landscapes of career-related digital sites and applications. For instance, Vigurs et al. (2017) observed that career information and interactive websites and on-line applications have mushroomed in different parts of the world. Vigurs et al. (2017) defined career websites as “on-line services that offer career information, including qualitative information such as career stories, diagnostic assessments or opportunities for interaction between individuals” (p. 1). The potential effects and benefits from using these diverse web-based applications are yet to be fully examined by research. The findings reviewed by Vigurs et al. (2017) suggested that positive outcomes from web-based interventions were contingent on how well they were connected to existing career services.

The most critical challenge to the development of career guidance ICT applications is how to make the best use of powerful digital technologies to create a system that could prepare individuals for transitions in a rapidly-changing and volatile environment (Hirschi, 2018; Tracey, 2020). Bimrose et al. (2015) argued that successful integration of ICT into career intervention services has much to do with three clusters of factors, which are policy support (e.g., government, organizational), workforce professional competencies, and the design of ICT applications that are fit for purposes. The focus of this paper is on the conceptual and design aspects of CACGS, even though we fully understand that policy and professional competencies are instrumental in realizing their positive effects. Career development professionals have to make use of their know-how in career guidance to design a technology system that is valid, reliable, personalized, and helpful to users. Specifically, we would like to achieve three objectives in this article. First, we would like to review the current status of CACGS and to identify their strengths and limitations. The review aims to capture the historical contexts where CACGS have been evolving in the past, present, and to the future, and how social changes have called for new frontiers to be developed. Second, we would like to explore features and components of future generations of CACGS. Specifically, we propose to use the Career Construction Theory (CCT; Savickas and Porfeli, 2012; Savickas, 2020) as a guiding conceptual approach, along with the trait-factor or person-environment fit models of career assessment which has been the dominant conceptual framework behind existing CACGS (Rounds and Tracey, 1990; Chartrand, 1991; Dawis, 2013). Third, using a recently developed CACGS in Hong Kong (the Infinity system) as an illustration (CLAP for Youth @ JC, 2019; Hong Kong Education Bureau, 2020; Leung, 2020a), we discuss system features and report initial findings that addressed the research question of whether using the system was helpful to students in developing their career adaptability, decision-making efficacy, and intention to pursue higher education.

## CACGS—Current Status

From a conceptual standpoint, past and existing CACGS are rooted in a trait-factor tradition of career assessment and guidance. The trait-factor approach was influenced by the early work of Frank Parson who proposed a career guidance approach consisting of the steps of helping individuals to (a) learn about themselves, (b) know the occupational world, and (c) choose occupations that matched with one's traits (Pope et al., 2019). Drawing from this approach, assessment tools were developed to assist individuals to make informed choices through understanding their traits and the world of work (Chartrand, 1991). The Minnesota Theory of Work-Adjustment (TWA) is one of the career development paradigms that was rooted in the trait-factor approach. Under the rubric of the TWA an array of career assessment instruments has been developed, including measures to assess career interests, values, and aptitudes (Dawis, 2013). The trait-factor approach is a conceptual predecessor of the person-environment fit (P × E fit) paradigm (Rounds and Tracey, 1990; Chartrand, 1991). The P × E fit paradigm still relies on having valid and reliable career assessment measures, yet humans are perceived as having the ability to make use of information to choose and shape his/her environment continuously to maintain and enhance congruence. Holland's (1997) theory of career interests is a case-in-point of the P × E fit paradigm. Holland's theory of career interests and personalities has a profound influence on career development and assessment, including the classification of interests and occupations that are embedded within CACGS. In sum, consistent with the trait-factor and/or P × E fit approaches, CACGS were developed with the tripartite objectives of (a) helping individuals to know more about themselves through using valid and reliable assessment tools (e.g., interests, values, and skills), (b) guiding individuals to explore occupations that are consistent with self-knowledge, and (c) learning the process of career decision-making and making informed choices.

The Computerized Vocational Information System (CVIS) was an early technology system connected to the career development theory of Roe (1956). Upon taking an interest inventory (the Kuder Preference Record) users were provided with a list of suggested occupations based on the occupational classification system of Roe along a spectrum of professional and skill categories. The CVIS was not an interactive system but it offered to users occupational information that were tied to their assessed career interest profiles (Harris-Bowlsbey, 2013).

The DISCOVER was an example of popular CACGS with a strong assessment component. Early versions of the DISCOVER system employed a digital version of Holland's Self-Directed Search (SDS; Holland, 1994). Based on the assessment results the system generated career and educational options for users according to the Holland RIASEC themes. In subsequent versions of the DISCOVER, the World-of-Work Map system (Prediger, 1976) and the UNIACT Interest Inventory (ACT Inc, 2013) were adopted but the model of Holland was still a part of the classification framework. The DISCOVER system has since evolved into a different web-based career assessment structure under the Kuder Career Planning System^®^ (KCPS; Kuder Inc., 2012; Harris-Bowlsbey, 2013). The KCPS has versions for different age groups, including sub-systems for elementary students, high school students, college students, and adults. Harris-Bowlsbey (2014) and McGrew (2018) outlined the range of positive outcomes resulting from using the KCPS, including improved career decision-making skills, career decidedness, successful transition to post-secondary education, academic success (e.g., higher GPA) and increased career planning motivation.

Another example of popular CACGS was the System for Interactive Guidance Information (SIGI) and the subsequent SIGI-PLUS developed by the Educational Testing Service for student and adult users. The SIGI system was designed with an emphasis on the assessment of individual values, exploration of occupational information, and use of an information processing model to predict career choices (e.g., use of criterion or aspects and subjective ratings to compare options and formulate desirable choices). The decision-making theory of Katz (1980) served as the conceptual guide of SIGI and its updated versions. SIGI-PLUS has nine different sections, which are: introduction, self-assessment, search, information, preparing, skills, coping, deciding, and next steps. The latest version of SIGI is SIGI-3, and it is offered on-line by the Valpar International Corporation (http://www.valparint.com). Evaluation findings supported the effectiveness of SIGI on a scale comparable to those produced by other career interventions such as career education and career counseling (e.g., Garis and Niles, 1990; Peterson et al., 1994).

The Making Better Career Decision (MBCD) was developed by the research team from Israel (Gati et al., 2003). The MBCD was an internet-based interactive career planning system based on the career decision-making difficulties framework of Gati et al. (1996). The MBCD guided users through a 3-steps process using a PIC model (pre-screening, in-depth exploration, and choice). The MBCD consisted of hundreds of occupations and users could make paired comparisons along 31 aspects or factors relevant to career decision-making (e.g., work values, preferred abilities, and work environment). Research findings suggested that users improved on career decidedness and they were satisfied with the process and alternatives identified. The MBCD has evolved into a web-based self-help system called the *Future Directions* website [www.kivunim.com; see Shimonia et al. (2019)].

Web-based CACGS at the national level have also been developed to facilitate career development of students and adults. A case in point of a national CACGS is the Occupational Information Network (i.e., O^*^NET), an on-line platform developed by the U.S. Department of Labor (U. S. Department of Labor, Employment and Training Administration, 2002). The O^*^NET is an open-access system for self-directed users. The information system of the O^*^NET is a digital, automated, and multi-media version of the Dictionary of Occupational Titles (U. S. Employment Service, 1991) where users could locate information on occupations classified according to interests, values, and skills. In addition, the O^*^NET^®^ career exploration tools offer self-directed assessment of interests, work values, and skills (Rounds et al., 1999). Through the on-line O^*^NET Interest Profiler, users receive an interest profile with explanations of the test scores. The information system and the assessment system of the O^*^NET platform are conceptually (i.e., P × E fit model) connected but they are standalone silos with no interactive capacity. Users could use the internal search function of O^*^NET to locate career information compatible with their assessment profile and search criteria.

Another example of national-level CACGS is the *myfuture* website of the Australia National Career Information Service (https://myfuture.edu.au/; Education Services Australia, 2020). The system is free and open for registered users in Australia and elsewhere. The key components of the *myfuture* website include on-line career assessment and profiling (interests, values and skills), information about occupations and career paths (e.g., employment information, skill levels and labor market information, career videos, career resources), as well as information about educational pathways in Australia (e.g., courses, institutions, subject-career connection). The *myfuture* website was developed with students as the primary audiences and student users could continue to use the service after leaving schools. Meanwhile, the *myfuture* website has information for parents, teachers, and career practitioners to equip them as supportive agents.

There are similar national-level career information and assessment platform, such as the National Career Service website of the United Kingdom (https://nationalcareers.service.gov.uk), and the MySkillsFuture website of Singapore (https://www.myskillsfuture.gov.sg/content/student/en/secondary.html). These national-level systems offer career assessment and occupational information specific to the social and labor contexts of the country. They are developed as part of the national career and educational strategies to support the career development of students and adults in schools and communities. Changes in national policies and strategies might affect how these platforms are sustained and supported.

### The Need for a New Paradigm for CACGS

The development of CACGS should be an important agenda of career development practice and research. CACGS could play a key role in engaging and serving the diverse needs of new generations of digital users. Digital applications are indispensable tools to sustain human and education services in an unpredictable world (e.g., COVID-19). Even though the literature on the use of ICT has increased quite rapidly in the last decade, given the fast-increasing use of technologies to support service delivery in educational and organizational settings, there is still a large gap between career development research and practice on one side and technology on the other that is yet to be bridged (Moore and Czerwinska, 2019).

The following are some critical limitations of existing CACGS at the private and national levels. First, career assessment for informed decision-making is the centerpiece of CACGS. A test-and-tell approach under the umbrellas of the trait-factor or P × E fit frameworks is insufficient in meeting the diverse needs of a new generation of users whose career development does not progress in a linear and predictable fashion. Most individuals must make multiple career decisions in their lifespans and have to cope with educational and career transitions that are not uniform. It is not to suggest that valid and reliable career assessment is not required, but CACGS have to formulate a new paradigm that allow users to explore and re-explore their careers, to discover and expand their interests and competencies, and to locate proactive actions to cope with rapidly changing landscapes.

Second, existing CACGS treat users as passive recipients of interventions designed by experts. New generations of CACGS should make use of new technologies to go beyond traditional career assessment to build new applications that encourage ownership and self-directedness. Developing CACGS capable of personalization is one way to promote ownership. A personalized CACGS should offer users a digital space where they could create their own account, view their test scores dashboard, and to track changes and progress. Moreover, a personalized system should have the capacity for users to keep career planning documents, make use of career planning search engines to explore, and receive feedback and notifications from the system that are tailored for their needs and preferences. Social media tools should be an integral part of personalized CACGS for users to connect and share with their social support networks.

Third, the positive outcomes of CACGS for users are not well-defined. The unifying objectives of most CACGS are self-understanding, career knowledge, and informed decision in the context of the P × E fit paradigm. These are important objectives but they are not sufficient. CACGS should further delineate outcomes in relation to attitudes (e.g., being proactive, readiness), competencies (e.g., adaptability, problem-solving), or actions (e.g., visit relevant workplaces). Outcomes could also be personalized based on the needs of users. A CACGS designed for continuous exploration should help users define and re-define career planning outcomes, monitor progress, with feedback loops to encourage resilience.

Fourth, CACGS should take advantage of the latest in computing technology to make the process fun for users. Gamification is one of ways to inject elements of fun into a learning system. Gamification involves the use of attributes (e.g., action/language, environment, game fiction) derived from games to applications and interactive environments that are outside the context of a game for the purpose of facilitating learning of attitudes and behaviors (Landers, 2015). Gamification has been widely used in the last decade to design learning and instructional activities in educational and organizational settings.

Fifth, CACGS should consider novel conceptual and measurement possibilities powered by technologies, including dynamic assessment, adaptive interpretation of assessment data, and use of big data and machine learning in the design of career assessment systems (Kalantzis and Cope, 2012; Tracey, 2020). These possibilities could greatly enhance the precision of measurement, personalization of interpretation, and accuracy of prediction from conventional and unconventional measures.

Sixth, CACGS are often silos that are disjointed from existing career guidance services and provisions in the users' context. For instance, Vigurs et al. (2017) reviewed a variety of career-related information, game-based, and interactive websites and concluded that the effectiveness of these career websites could be enhanced if they were connected to the existing career support service and programs in the contexts of the users. Hence, CACGS should connect and empower users to use support service and networks in their communities.

Last but not the least, career services are not well-equipped and prepared to use CACGS in service delivery. The lack of readiness on the part of service providers were key findings from a study by Kettunen and Sampson (2019). Participants from 16 countries were asked to share their perceptions about the use of ICT in their regions and the challenges that they were facing. Key findings on challenges included: (a) not having sufficient access to ICT in career services (e.g., inadequate ICT infrastructure), (b) lack of updated career information to complement ICT systems, (c) lack of professional skills and competencies among staff on using ICT, (d) and insufficient integration between ICT and existing services.

In the past decade, we have witnessed tremendous social and economic changes around the globe propelled by technologies. These changes have come to be known as the 4th industrial revolution (Schwab, 2016) and it has fundamentally altered all aspects of work and life, including how businesses and organizations are operated, and how individuals learn and work, plan their future, and seek help from professionals. Hirschi (2018) and Lent (2018) observed that the 4th industrial revolution has made tremendous impact on career development. Examples of these impact included personal distress and social disconnection, reduction in job security, automation and work displacement, emergence of new forms of employment (e.g., self-employment), and the development of virtual platforms for individuals to work and learn collaboratively. New generations of workers have to face novel challenges, such as unconventional career pathways, a lack of work-life balance, and having to integrate multiple identities emerging from diverse work and life roles. CACGS should undertake a new paradigm to equip users to cope with these changes.

In addition to changes driven by technological advances, there have been many instances of unpredictable social changes and challenges threatening human well-being (e.g., natural disasters, political instability). The COVID-19 global pandemic is a case-in-point of how unpredictable and complex events could push work, education, and life into a “new-normal” mode, and how technologies could become a part of the social and work structure in the new-normal (Fouad, 2020).

Against the above backdrops, there is considerable room for innovative research and development work on the use of ICT and to push for a new frontier in career development interventions. CACGS should be fully integrated into the delivery of career services in educational and organizational settings to level-up service flexibility, especially when resource support might not sustain the full spectrum of traditional services in the “old normal” (Bimrose et al., 2011).

## Career Construction Theory and CACGS

The Career Construction Theory (CCT; Savickas, 2013, 2020) was built on the premise that the increasingly unpredictable and fast-transforming social and economic contexts have called for a new paradigm of career development. According to the CCT, the dominant paradigms of career development in the past decades such as the P × E fit paradigm were not developed with the new global realities in mind. These models seek to match individuals with compatible work environments and to guide them for transitions across predictable life and career stages, as opposed to managing careers in a globalized context where variations in individual work experiences and career dis-continuities and fragmentations are the norms rather than exceptions. The CCT offers fresh perspectives and a new paradigm to guide new generations of CACGS. In this article, three aspects of the CCT that are most relevant to the development of CACGS are discussed, which are the narrative tradition, model of career adaptability, and life-design interventions.

### Narratability and Career Stories

CCT is anchored on a social constructivist perspective (Young and Collin, 2004; Patton and McMahon, 2017) that construes development as driven by adaptation and continuous self-construction. Individuals have to engage in an active process of meaning making and re-making to navigate through career development tasks and challenges. A narrative strategy is used by social constructivist approaches in which words, language, and story-telling are channels of meaning and identity construction (Savickas, 2013). Human and therapeutic interactions are means for individuals to use narratives “to facilitate self-reflection and elaboration of self-concepts toward an enhanced self-understanding which is subjectively and contextual truthful” (McIlveen and Patton, 2007, p. 228).

CCT maintains that subjective meanings of work and careers are expressed through a set of themes that could be extracted from past memories, present experiences, and future aspirations. Career and life themes are key components of an open-ended career story where life/career experiences and choices are weaved together into a meaningful whole (McMahon and Patton, 2017). The subjective career and life themes are not static but are evolving narratives of the self that serve to guide, regulates, and sustain career choices and behavior.

Career interests, skills, and values as unique characteristics that are co-constructed by the self and the society. Interest and personality typologies are not representations of the realities but are merely one's “resemblances to socially constructed clusters of attitudes and skills” (Savickas, 2013, p. 154). At the practice level, career construction interventions make use of career typologies and resemblances as self-construction strategies to facilitate career exploration, yet the purpose is not to fit individuals with heterogeneous potentials and self-concepts into compartments of types and sub-types, or to treat these constellations of preferences as stable traits that could predict the future. Career construction involves a much broader process of building self-identity and test scores are treated as references. McMahon et al. (2020) illustrated how career stories could be integrated with career assessment. The authors used a story-telling career interview strategy to deepen exploration of interest scores. Through encouraging clients to narrate career stories from their experiences related to measured career interests, the structured counseling process fostered understanding of career themes through connectedness (e.g., client-counselor), meaning-making, agency, learning, and reflection.

The narrative propositions of CCT offers an array of implications for the development of CACGS. For instance, career assessment and career stories are not mutually exclusive interventions but rather complementary processes that could enhance the cumulative effects of the respective approaches (e.g., McMahon et al., 2020). A digital system should include tools that can help individuals to construct and generate insights from their career stories. Career assessment results in the context of the CCT are observations that could inform life themes and enrich career stories in the process of meaning-construction.

### Model of Career Adaptability

CCT views career adaptability as the bridge between the self and society or the person and environment. The career adaptability model of CCT has four inter-related components or strategies which are adaptive readiness, adaptability resources, adapting responses, and adaptation results (Savickas and Porfeli, 2012; Savickas, 2020). Adaptive readiness refers to personal characteristics of readiness or willingness to engage in goal-directed activities to cope with career development tasks and transitions. Adaptability resources is the resourcefulness of a person to solve complex yet unfamiliar career challenges. Resourceful individuals use four psycho-social strategies to regulate career development which are: (a) concern—being concerned about one's future role as a worker, (b) control—striving for personal control over one's career future, (c) curiosity—being curious in exploring future scenarios and self-possibilities, and, (d) confidence—having confidence to pursue career and life aspirations (Savickas, 2020). Adapting responses are the actions taken to cope with career transitions, such as orienting, exploring, deciding, planning, and problem-solving. The development of readiness traits and resourcefulness along with actions taken should lead to positive adaptation results such as career success and satisfaction.

The adaptability model of the CCT serves as a useful guide to develop future digital CACGS. Most importantly, the goals of CACGS should be set on empowering readiness and willingness in career planning, to make use of one's resourcefulness to generate internal and external support (including social support), and to problem-solve career transitions (e.g., understand and take adapting actions) with resilience.

### Life-Design Interventions

Life-design interventions could be viewed as the intervention component of CCT (Savickas et al., 2009; Hartung, 2019). Life-design interventions are outcomes of years of conceptualization and practice efforts by career development scholars internationally to use personal constructs, biographic hermeneutics, and narrative paradigm in career interventions (McMahon et al., 2005; Chen, 2011; Nota and Rossier, 2015; Hartung, 2019). Life-design interventions views individuals as proactive meaning-making agents searching for an answer to the key question of “what purpose does work serve in my life?” In order to identify the purposes, life-design interventions cultivates narratability, or the capacity of a person to tell his/her own life story through weaving together meaningful themes from past experiences and future aspirations.

Life-design interventions involves the steps of: (a) career construction where clients narrate small stories from life episodes, events, and incidents; (b) career deconstruction and reconstruction where stories or micronarratives are re-conceptualized and re-configured into a meaningful identity narrative or life portrait; and (c) career co-construction in which the client and counselor work together to “edit” the life portrait and the identity narratives, to place the problem in a new story, to cultivate action intention, and to actualize that identity in the real world (Savickas et al., 2009; Savickas, 2012). Reflexivity is an important process skill. Life-design conceptualizes reflexivity as “careful consideration of current issues that produces a new perspective to guide life choices” (Savickas, 2016, p. 84). Reflexivity ignites insights and re-integration that are instrumental to changes and agentic actions.

Life-design offers future CACGS with rich opportunities for novel development. For instance, in the language of life-design intervention, the process of using CACGS should shape narratability, adaptability, intentionality, and reflexivity, in addition to helping users to plan and manage their careers and career identity across the lifespan. CACGS could create digital platforms with connected and graded learning experiences where meaning and career construction skills could be acquired and put into practice.

### Summary

CCT offers CACGS a fresh paradigm to understand career development in an age of rapid changes and discontinuities. Developing skills for meaning and career story construction, strengthening career adaptability, and empowering adapting actions are examples of propositions from CCT that could inform new generations of CACGS. In the following section, we describe a CACGS developed in Hong Kong for secondary students that sought to transform some of the CCT conceptualization into a set of connected digital tools and applications to foster career development.

## Infinity—Career Planning and Management System

A digital career development and planning system called “Portfolio Infinity: A Career Development Self-learning and Management System” (hereby referred to as the Infinity system) was developed in Hong Kong for senior secondary students (CLAP for Youth @ JC, 2019). The Infinity system was developed as part of a funded project to support the career guidance activities of secondary schools (Leung, 2020a). The system was adopted by the Hong Kong Education Bureau for all secondary students in the City [see Hong Kong Education Bureau (2020) for the user manual], and the system was renamed “My Life Planning Portfolio” (https://portfolio.lifeplanning.edb.gov.hk/index.php?lang=english).

Hong Kong did not have a CACGS for secondary students and schools relied on scattered career development and assessment materials from printed and internet sources (Ho and Leung, 2016). There is a need to develop a CACGS to augment career guidance activities and to provide tools that students could use. The development of the Infinity system involved the collaboration of professional teams in career development practice, computing technologies, and research. It also involved close partnerships with schools, universities, and the funder. In this section, the following themes are addressed: conceptual underpinning, methodology, applications and functions, and user outcomes.

### Conceptual Underpinnings

The Infinity system is composed of qualitative and quantitative applications that are inter-connected, designed to guide users to construct and re-construct their career stories, and in the process learn meaning construction skills, in accordance with the propositions of the CCT reviewed in the preceding section of this article.

In addition to the CCT, the Infinity system also referenced the career self-management model of Lent and Brown (2013). As an extension to the social-cognitive career theory (SCCT), Lent and Brown (2013) proposed the career self-management model emphasizing career preparedness at a time of rapid changes and uncertainties. Lent and Brown (2013) defined adaptive career behaviors as “behaviors that people employ to help direct their own career (and educational) development, both under ordinary circumstances and when beset by stressful conditions” (p. 559). Adaptive career behaviors include tasks that are relatively normative across developmental periods, as well as coping skills and processes to manage transitions and unforeseen career challenges. Lent and Brown (2013) noted that adaptive career behavior and career adaptability in the CCT framework are complementary constructs in that both concepts address functioning, resilience, self-regulation, and resourcefulness in response to changes and transitions.

The Infinity system was informed by the career self-management model of Lent and Brown (2013) in at least two ways. First, the applications of Infinity are developed to encourage self-ownership including a career self-management mindset. Second, the Infinity system referenced the adaptive career behaviors from Lent and Brown (2013) to form a list of career planning actions for users to consider. In the Infinity career education curriculum, students are encouraged to identify actions to take in each of the lessons (e.g., a personalized action list), and career teachers could follow-up on those actions in class or during the personal career guidance sessions. The process is aimed to strengthen career preparedness.

The Gatsby benchmarks from the United Kingdom are also used as references (Gatsby Charitable Foundation, 2018). The Gatsby benchmarks specified eight dimensions or benchmarks of “good career guidance” in secondary schools developed from good-practice research findings in several countries. The benchmarks are: (i) having a stable career guidance program, (ii) opportunities to learn from career and labor market information, (iii) career programs addressing the needs of each pupil, (iv) subject curriculum learnings are linked with careers, (v) opportunities to meet and interact with employers and employees, (vi) engagement and experiences at workplaces, (vii) opportunities to visit and learn about further and higher education, and (viii) receiving personal guidance. The Gatsby benchmarks provide important anchors on what constitute adaptive career actions for secondary students from a school programming perspective.

Meanwhile, Brown et al. (2003) and Brown and Ryan Krane (2000) identified five critical ingredients of career interventions, which are workbook and written exercises, individual interpretation and feedback, world of work information, modeling, and attention to building support. These elements are considered and synthesized into the Infinity system (e.g., narrative exercises, information functions, and social communication capacity). Several desirable ingredients noted by Brown et al. (2003) were also considered, including values clarification, card sort procedure, and personal performance accomplishments.

### Methodology and Objectives of Infinity

We referenced the on-line learning design determinants of Lehman and Conceicao (2010) and Bimrose et al. (2015) in building the Infinity system. The design determinants are: types and focus of content, format of learning experience, interactive strategies, role of facilitator, type of technology being used, and kinds of support. The methodology of building Infinity involved engaging professionals to develop fit-for-purpose learning experience and content, create channels of interactions for users and supportive networks, connect users to career guidance services in school settings, and forge close collaboration between cross-professional teams and stakeholders to ensure that right technologies and methodology were employed. The following steps summarized the methodology that was followed in building the Infinity system, including the development of system objectives.

1. *Need analysis*—A need analysis was carried out in 2014 to understand the career develop needs of students and school programs from the perspective of career teachers. The most pressing needs included a lack of tools, resources, and assessment materials for diverse students, student passivity, insufficient professional competencies, lack of support from school leaders, and inadequate curriculum materials for career education (Ho and Leung, 2016).
2. *Envisioning and objectives development*- At the initial phase of the project in 2015 to 2017, a process of envisioning was carried out to understand and align the objectives of the project including to envisage how the Infinity system would look like when it was completed. Five secondary schools participated in the process as pilot schools and teachers and school principals from these five schools were closely involved in the envisioning of Infinity from objectives to the final production. The following were the objectives of Infinity agreed upon from the envisioning process: (a) create an all-in-one and user-friendly digital platform with inter-connected and interactive functions for career construction, (b) offer tools for users to develop their career plans and facilitate informed decisions, (c) create a digital career education platform with relevant and connected curriculum, (d) define adaptive career behavior and career development outcomes, (e) develop a dynamic career information search engine, (f) build a fun and engaging system for users to use continuously, (g) connect social support systems, and (h) provide schools with aggregate data about students to inform career guidance programing.
3. *System architecture, system security, privacy, and user journey optimization*- Technical vendors were engaged to specific system architecture, to assure that the system met the latest security and privacy benchmarks, to develop a convenient and secure sign-on system, and to create an engaging user journey (e.g., user-friendliness, use language that users could relate to).
4. *Milestone development*- Project management teams were formed to monitor progress related to the different applications of Infinity. Software engineers and programming staff worked on building the front- and back-end of the system with collaborations from vendors.
5. *User Acceptance Testing (UAT)*- Before each application of Infinity was rolled out for service, UAT was carried out and users (e.g., teacher and students) were asked to test-use the application to identify technical and user journey gaps.
6. *System roll out*- When an application is rolled out the project professional team would conduct training for career teachers who were considering to use the application. Only project partner schools were given access to using the application. The project engaged schools in two levels of partnerships (those with intense on-site professional support and those with only limited district-based professional support and the use of the digital platform). Due to the availability of training and support from the project, career teachers from partner schools should be well-equipped to support students in using these applications.

### Learning and Organizational Framework, Applications, and Functions of Infinity

**Figure 1:**
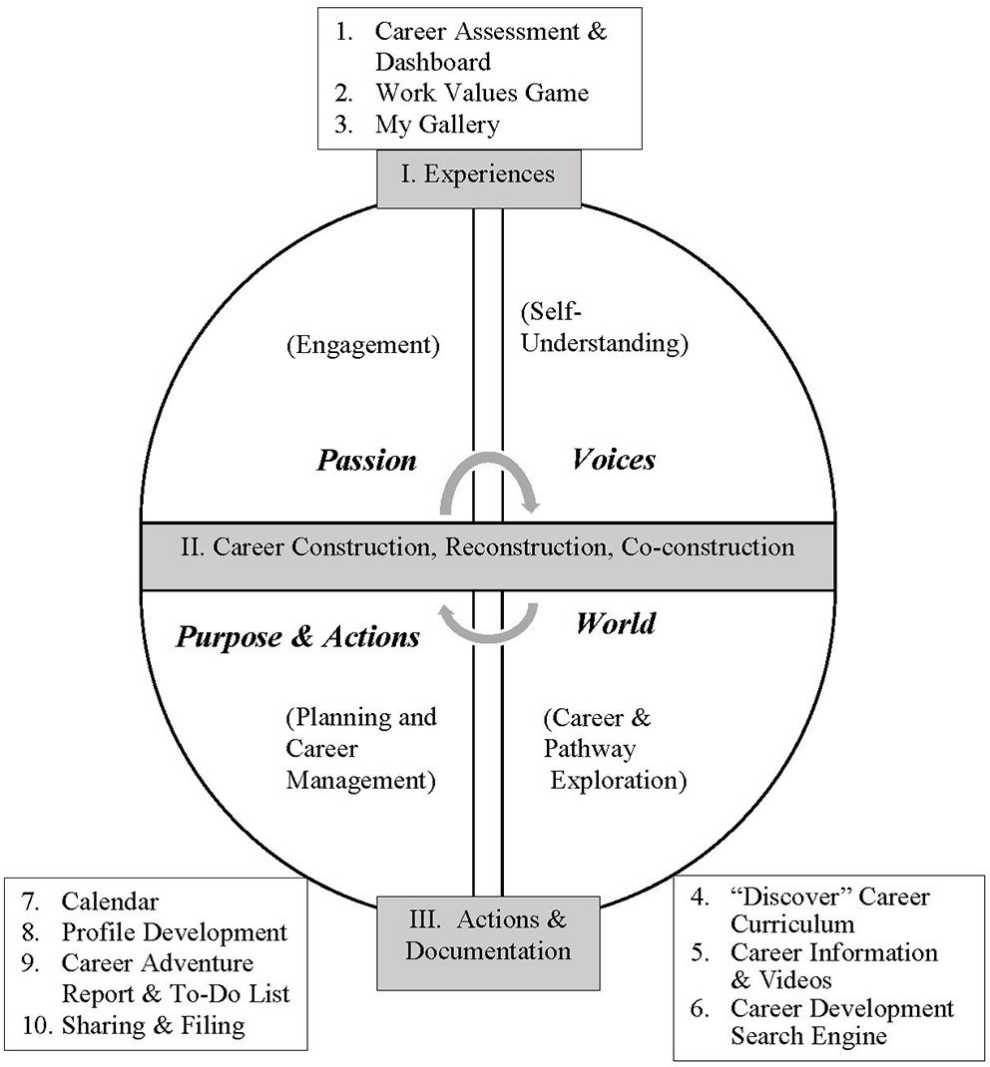
The learning and organizational framework of the infinity system^a^: career planning steps and career construction^b^. ^a^Infinity applications are numbered from 1 to 10. Please refer to Table 1 for description of the functions of the applications. ^b^The CCT learning process is indicated in the shaded boxes.

The applications of Infinity are organized under a learning and organization framework (Figure 1) consisting of the steps of career planning and the career construction process. The steps of career planning are: (a) engagement (“passion”), (b) self-understanding (“voices”), (c) career and pathway exploration (“world”), and (d) planning and career management (“purpose and actions”). To avoid jargons that users might not understand, the four interactive career planning steps are also named passion, voices, world, and purpose and actions, respectively, for easy comprehension and empowerment. These career planning steps aim to communicate to users the process it takes toward developing purposes and taking meaningful actions. The steps coincide with the career development tasks of young people in the exploration stage of career development according to Super's career development theory, which are crystallization, specification, and implementation (Super et al., 1996). Engagement is a prerequisite in career interventions (Amundson, 2017), and in the Infinity system it is viewed as an overarching step that propel users to take on other career planning steps. The four steps can be viewed as a circular experience in which the student could return to and renew the learning process whenever there is a need. The Infinity applications associated with each of the above steps are summarized in Figure 1 and Table 1, but it should be noted that the applications could be used in multiple steps of the above learning journey.

**Table 1:** Applications and functions of the infinity system.

**Applications**	**Brief description of functions**
1. Career Assessment and Dashboard	• *On-line system*: Career assessment could be done on-line and reports explaining the results are sent to users and career teachers (with permission) immediately upon completion. Career assessment test scores are based on local norms. • *Personalized and visual display*: Career assessment scores are personalized and visually displayed in a dashboard under the account of each user. The visual presentation allows users to comprehended their career interests and readiness easily.
2. Work Values Game	• *Gamification in work values assessment*: The Work Values Game is an educational application. A total of 20 work values are included in the game-based application. Users are asked to rank order the work values based on how important they are in choosing one's future work. The gaming environments allow users to integrate gaming, learning, and career exploration.
3. My Gallery	• *Use of multi-media materials in career assessment*: The use of visual and pictorial representations is a major strategy in qualitative assessment. My Gallery allows users to express their thoughts and feelings on career development *via* multi-media modalities (e.g., pictures, collages, emoji, and videos).
4. “*Discover*” Career Curriculum	• *Career education*: The career development curriculum has 12 lessons. The lessons are inter-connected and organized thematically to optimize learning. Lessons are conceptually based on the CCT, and they introduce students to all the applications of Infinity for the purpose of constructing their career stories. User guides are developed for instructors using the curriculum with detailed explanations and power-point slides. • *On-line flexibility*: Interactive and digital worksheets are developed and students/users could submit their completed tasks and assignment on-line *via* the Infinity system.
5. Career Information and Video	• *Concise career information*—Career videos feature workers of new, popular, and emerging careers. There is a set of career briefs (200+) thematically organized based on Holland's framework. The briefs consist of general information about careers for easy consumption (e.g., brief descriptions, educational requirements, compatible skills and interest, and information links). The career information is also linked to the career and educational information system of the Education Bureau.
6. Career Development Search Engine	• *Database—The search engine is equipped with a large database consisting of career- and education-related sites in Hong Kong, Asia, and internationally*. • *Search results, related search terms, search history—Users can provide one or multiple keywords and the engine returns relevant results determined by the ranking function from the index available. Related search terms and keywords are provided to the users when they are using the search engine. History of past searches are displayed*. • *Ranking and filtering—The ranking and filtering functions are designed to optimized searches through matching with relevance, recentness, rating, and popularity*.
7. Calendar	• *Time reminders and alerts—The Calendar application of Infinity sends alerts to users on upcoming events and messages received at the account (e.g., from teachers). Users could put upcoming tasks on the calendar and the system could send them alerts at the scheduled date/time*.
8. Profile Development	• *Shine ∙ My Profile—A simple to use profile for users to construct their career stories, including sections on interest, values, leisure, functional skills, and career and life goals. Multiple designs are available for users to choose*.
9. Career Adventure Report and To-Do-List	• *Define adaptive actions—The Career Adventure Report consists of a total of 22 actions that are organized under the steps of engagement, self-understanding, career and pathway exploration, and planning and career management. Users could add personalized tasks to the list (i.e., the to-do-list). Users are asked to input when the tasks have been completed. Gamified check marks are inserted by the system to indicate completion. The calendar could send user alerts on upcoming events related to planned tasks*.
10. Sharing and Filing	• *Social support and documentation—Documents and reports generated from the Infinity system could be shared with teachers, peers, and parents. A virtual storage space is assigned to users to keep and organize their files. An account management system is developed to target the complexities of class-based system in secondary schools*.

The learning and organization framework of the Infinity are connected to the CCT career construction process (Figure 1). The process involves experiences, construction and reconstruction, co-construction, actions, and documentation. The Infinity applications (e.g., career assessment and dashboard, career information and videos) offer users an engaging career development experiences, and the experiences trigger a desire to proactively create and articulate one's unique career stories and career and life themes. Career construction happens when the students are involved in transforming what they learned from experiences into observations, themes, and mini-stories (e.g., “Shine • My Profile,” “Discover career curriculum”). The process could refine and update students' understanding of self and the world (re-construction). The social and sharing functions of the Infinity system (e.g., with peers, teachers, and parents) along with class- or group-based processes could encourage the use of contextual resources to strengthen students' career planning endeavors (i.e., co-construction). The system encourages users to take purposeful actions in their contextual reality to problem-solve their career transitions (Table 2). In addition, the applications and functions of the Infinity system could shape an array of attitudes and competencies specified by the CCT. For instance, the applications under self-understanding could strengthen adaptive readiness, narratability, intentionality, and reflexibility, the applications under career and pathway exploration could strengthen adaptability resources, and the applications under planning and career management could define and encourage adapting actions. The Infinity system offers users documentation tools to file, archive, and modify their career planning findings and observations.

**Table 2:** Adaptive career actions from the career adventure report of the infinity system.

**Career planning steps**	**Adaptive actions**
Engagement	1. Participated in __ activities or event (in or outside school) connected to your career and life interests^e, g^ 2. Talk with alumni or seniors to learn from their experiences and possibilities for my future^e, g^ 3. Participate in career and life planning classes/lessons^a, d^ 4. Engage in activities to develop my interests^c^ 5. Continue to be involved in community or paid work^f^
Self-Understanding	6. Take a career interests test and understand the results^a^ 7. Identify ___ career options that you are interested in^f^ 8. Identify ___ further study options that you are interested in^g^ 9. Review experiences and identify ___ skills that you are proud of^c^ 10. Consider career pathways that match with personal interests and characteristics, and ways to pursue them^d, f^
Career and Pathway Exploration	11. Talk to ___ people who are working in careers or fields that you are interested in^e^ 12. Talk with ___ students or alumni who are studying in programs you are interested in to get information about the programs and career prospect^g^ 13. Visit ___ local higher education institutes (e.g., universities, institute of vocational education)^g^ 14. Conduct information search related to study programs or career options of interests (e.g., labor market information, job requirements, career prospects) and consider how they fit with self^b, d^ 15. Visit ___ workplaces (e.g., companies or enterprises) that are connected to your career interests^e, f^ 16. Participate in taster programs on further study or workplace^e, f^
Planning and Career Management	17. At least once a year, talk with your teacher or parents/ guardians about your progress and needs related to career and life planning (e.g., dreams, difficulties, direction)^h^ 18. Participate in a mock interview training (for further studies or employment)^b^ 19. Know my needs and be prepared for the transition to my next destination (further study or workplace)^c^ 20. Actively seek out people and community support as well as opportunities to help me achieve my career and life goals^g, h^ 21. Can balance my study/work and life effectively^c^ 22. Learn how to construct a personal CV or career development profile^b^

*Adaptive career behaviors are mapped with the Gatsby good career guidance benchmarks*.

a *(i) Having a stable career guidance program*.

b *(ii) Opportunities to learn from career and labor market information*.

c *(iii) Career programs addressing the needs of each pupil*.

d *(iv) Subject curriculum learnings are linked with careers*.

e *(v) Opportunities to meet and interact with employers and employees*.

f *(vi) Engagement and experiences at workplaces*.

g *(vii) Opportunities to visit and learn about further and higher education*.

h *(viii) Receiving personal guidance*.

The “Discovery” career education curriculum illustrates how the applications of Infinity are tied to the above CCT-based learning process. All the applications of Infinity are used to structure the learning experience in the career curriculum. The career curriculum consists of 12 thematic lessons and two of the sample lessons are summarized in Table 3 to illustrate how the Infinity tools are used to facilitate the CCT-based learning framework (Figure 1). In each thematic lesson: (i) students engage in activities and experiences led by the career teacher, (ii) make use of worksheets and applications of Infinity to construct and re-construct career stories, (iii) complete a “footprints” worksheet which is an in-progress summary of career stories and themes identified and actions to take, (iv) share their “footprints” with peers and teachers, and (v) document their materials so that could review and edit their stories, findings, and reflections.

**Table 3:** Examples of how infinity applications are used in career construction through the career education curriculum.

**Example of theme**	**Experiences**	**Construction/re-construction**	**Co-construction**
Lesson on Work values	•Share a favorite “motto” or “saying” with fellow students, including why it is important, and how it affects his/her career planning (a career story) •Learn about different work values categories from a short presentation •Watch a short video from the “Infinity career information” application illustrating the work values of a worker—A self-employed musician operating a studio (a career story of a role model)	•Work on a short checklist with items related work values categories to have an initial exploration of personal work values •Record findings, observations, and reflections on a worksheet related to work values checklist (i.e., enrichment of career story)	•Play the “Work Values Game” in the Infinity system and share with peers and teachers their findings and reflections •Work in small groups to view suggested websites related to occupations of interests to members (use of Infinity search engine), and identify work values of workers. Students have to complete a group and a personal reflection on the “Footprint” summary which is an in-progress record of their reflections and insights (i.e., collection of career stories)
	•Facilitate adaptive actions – #2 and #11 of Table 2	•Facilitate adaptive actions – #6 and #10 of Table 2	•Facilitate adaptive actions – #6, #10, #14, and #19 of Table 2
Lesson on Developing my career planning “to-do list”	•The “8” possibilities exercise—Engage students to identify and share different computations that could arrive at a solution of “8” (e.g., to illustrate that there are many possibilities and pathways to achieve an objective) •Share a “traffic-light” model of career goals status with examples to track goal attainments (e.g., green=goals achieved but can set new ones) •Share a time- and career-management model with examples—identify task urgency and importance •Watch a short video from the career videos collection to identify examples of career planning actions	•Review the career goals set in the previous lesson and use the “traffic-light” model to assess progress and to “edit” them if needed (e.g., re-construction) •Use the actions in the career adventure report as examples and to create students' personal career to-do-list in the Infinity system, to use the calendar application to set the date/time for tasks to be completed •Use a worksheet to place actions from to-do-list on the time management matrix to assess urgency by importance, and plan the way-forward •Record the to-do-list in the “Footprint” summary	•Work in small groups to share goals and their career to-do-list •Share his or her to-do list with teachers and teachers could give feedback •Encouraged to share and discuss the to-do-list with parents or other key social supporters
	•Facilitate adaptive actions – #2 and # 19 of Table 2	•Facilitate adaptive actions – #19 and #21 of Table 2	•Facilitate adaptive actions – #19 and #20 of Table 2

The career assessment and dashboard applications offer users the option of taking four assessment tools, which are the Career Interest Inventory (Leung, 2020b; Leung et al., 2021), the Basic Interest Markers (Liao et al., 2008), the Career Decision-Making Difficulties Questionnaire (CDDQ; Leung et al., 2011), and the Career Adaptability Scale (Hou et al., 2012). Local norms are used in score interpretation and profile presentation. The assessment report sent to students provides a narrative explanation of scores. Teachers have access to a user and technical manual with information about the assessment tools, including test scores, psychometric properties, interpretation, and ethical and professional guidelines (Leung, 2020b).

The Work Values Game (WVG) is a case-in-point of a gamified application in career development. Values clarification has been viewed as an important ingredient of career interventions (Brown et al., 2003) and the CCT also identifies values as a key element to extract from career stories (Savickas, 2012, 2014). Instead of adopting a quantitative measure of work values, a cord-sort approach consistent with the constructivist process was used (Parker, 2017). Users (or players) could connect their salient work values to career options and stories. Playing the WVG consists of the following steps:

a. The player has to choose a character and then play a jumping-game in which the chosen character has to jump and maneuver upward using the mouse pad, and to score points and collect “gems” labeled with different work values.
b. Upon scoring sufficient points, the player is presented with a “wheel of fortune,” and after rolling the spinning wheel a worker would emerge (e.g., furniture designer), and the user would be asked several questions on work values that are important to this worker. After correct answers are identified, they have to continue with the jumping game to collect more points and work value gems.
c. After several rounds of encounter with different workers, the player moves on to the work values sorting exercise. The player has to first identify the top-10 values from the list of 20 work values, and then to sort out the top-5 values in sequence.
d. After confirming the rank-order of work-values, the system generates a report to the player which describes and explains the 20 work values and how they are important to career choice and satisfaction. The report also summarizes the top-ranked work values of the player.

The WVG is an educational game for users to explore and understand work values. There is no test score and the results are qualitative. Players learn about how diverse work values are connected to different occupations. They also identify the work values that are most important to them. The sample career education lesson in Table 3 illustrates how work values exploration are structured using the tools of Infinity including the WVG.

Table 2 summarizes the set of adaptive actions included in the “career adventure report” of the Infinity system. The list is meant to be career planning actions that are “typical” to senior secondary students. Students could review their progress on completing the “expected” career development tasks as partial outcomes in their secondary school years. We made references to adaptive career behaviors identified in Lent and Brown (2013), as well as the eight dimensions of good career guidance from the Gatsby benchmarks (Gatsby Charitable Foundation, 2018), and generated a draft of adaptive actions. We consulted career teachers from partner schools, including teachers from the five pilot schools who were involved in the envisioning of Infinity on the salience and relevance of the identified actions. The process resulted in 22 actions that are organized along the steps of engagement, self-understanding, career and pathway exploration, and planning and career management, similar to how the applications of Infinity are organized. The 22 adaptive actions also encompass the eight aspects of the Gatsby benchmarks, and completions of the actions should facilitate schools to meet career guidance standards. In addition to the suggested adaptive actions, users could add personalized tasks to the list to make up a “to-do-list” of their own. The mapping between the Infinity conceptual underpinnings and the adaptive actions should enhance the efficacy of actions taken by users toward achieving positive outcomes from using the system.

In sum, the Infinity system is an innovative CACGS designed for users with different career development needs. The CCT is the conceptual thread that connects and unifies the applications of the system. Users could learn, explore, and construct their career stories and plans continuously on their own or with the support of teachers and counselors.

## Evaluation of the Infinity System

### Research Questions of Study

The major research question addressed by this research was whether using the Infinity was helpful to students. In the context of the CCT, this research question was further refined into three sub-questions, which were: (i) whether using the Infinity system helped to develop students' adaptability resources, (ii) promoted career decision-making efficacy and clarity, and (iii) motivated students to consider pursuing higher education. Students who used the Infinity system were compared with those who did not. We used measures that are core to the adaptability model of Savickas and Porfeli (2012), which are the career adaptability scale (CAAS; Hou et al., 2012; a measure of adaptive resources) and the career decision-making difficulties questionnaire (CDDQ; Leung et al., 2011; a measure of adapting responses) and a career decision clarity measure (Gati et al., 2001). In addition, the Infinity system was designed to facilitate career education and educational aspirations, we used university education aspirations and understanding of career planning as measures to gauge potential benefits in education. Intervention studies based on the CCT have used adaptability resources and adapting responses as outcomes (e.g., Cheung and Jin, 2016; Cadaret and Hartung, 2020) as opposed to including measures covering all the four constructs of the adaptability model, which is important if the research objective was to examine model validity.

The following hypotheses were examined:

H1: Students who used the Infinity system should report a higher level of adaptability resources than students who did not. The differences between the two groups should be found the four subscales of the CAAS.
H2: Students who used the Infinity system should display lesser career decision-making difficulties than those who did not. Differences between the two groups should be found in the three subscale scores of the CDDQ (H2a). Relatedly, the user group should express stronger career decision clarity than the non-user group (H2b).
H3: Students who used the Infinity system should be more inclined toward pursuing university education, including local government-supported universities and other university options (e.g., cross-border options) than students who did not (H3a). In addition, they should report clearer understanding of what was career planning than the latter student group (H3b).

The Infinity system was built to facilitate the career development of all students. In each of the above hypotheses, we presumed that the effects should be the same across genders and students from schools of different academic backgrounds (i.e., school banding). Accordingly, gender and school banding were treated as independent variables in testing the respective hypotheses.

### Participants and Procedure

Participants included 2,935 12th graders (1,549 females and 1,374 males, 12 unidentified) who enrolled in schools that participated in a career development intervention project in Hong Kong [“CLAP for Youth @ JC” project, see Leung (2020a)]. The data of this study were collected at the end of the 2019–20 school year. Participants were asked to complete a survey when they exited high school and there was an item asking whether they had used the career exploration and assessment of the project (i.e., Infinity), with 844 indicated they had, and 2,091 indicated they had not. Analyses on the helpfulness of the Infinity system were based on the responses of these two groups (i.e., users and non-users).

The mean age of participants was 17.40 (sd = 0.81), and they came from 49 secondary schools representing the full spectrum of academic achievements. Academic achievement of schools in Hong Kong was classified roughly by a school-banding system, and 19.2% of participants of this study came from band-1 school (i.e., high achievement), 48.7% from band-2 schools (medium-achievement), and 32.1% from band-3 schools (i.e., low achievement). Secondary schools participating in this project were eligible to receive professional support services from the project team to strengthen career guidance service, including the use of the Infinity system. The use of the Infinity was optional, and schools could choose professional support from the project team based on their needs (e.g., work-place attachment and learning, individual career counseling, parent education, school-based curriculum). The research activities of the project, including all the data collected for this study, were reviewed and approved by the research ethics committee of the author's institution. School, student, and parental consent were obtained prior to data collection, and steps were taken to ensure that established ethical protocols for data collection and security were followed.

The exit survey (paper-and-pencil format) was collected from schools who consented to participate in the research (surveys were administered around February to May 2020). All the measures reported in this study were included in the exit survey so that students could complete at one time. As an incentive to school participation, the research team summarized findings from the exit survey and a report was sent to each of the participating schools to inform school improvement. The Infinity system was conceptualized and built during the period of the project (2015–2020), and applications rolled out for users at different time periods. The system was completed at the beginning of the 2019–20 school year (i.e., September 2019) and 12th graders who graduated from the 2019–20 school year were most likely to have used the multiple applications of the system during their study period.

### Measures

Students completed the exit survey which contained all the measures used in this study. Measures used in this study are summarized below.

#### Career Adapt-Abilities Scale (CAAS)

The Chinese language version of the CAAS international form (Hou et al., 2012; Savickas and Porfeli, 2012) was used to measure adaptability resources. The CAAS has four subscales, which are concern, control, curiosity, and confidence. Examples of items are: “thinking about what my future will be like” and “making decisions by myself.” A 5-point scale was used from 1 (not strong) to 5 (strongest), with higher scores denoting higher levels of adaptability resources. In this study, the CAAS short-form (Maggiori et al., 2017) was used with three items on each of the four subscales. Maggiori et al. (2017) reported an overall alpha of 0.90 for the CAAS short-form, and the alphas for the four subscales ranged from 0.77 to 0.83 (French version).

#### Career Decision-Making Difficulties Questionnaire (CDDQ) and Career Decision Clarity

The CDDQ (Gati et al., 1996; Leung et al., 2011) was used to measure adaptive responses or decision-making efficacy. The CDDQ has 34 items covering three dimensions of career decision making difficulties, which are readiness, lack of information, and inconsistent information. Example items are: “I know that I have to choose a career, but I don't have the motivation to make the decision now” and “I find it difficult to make a career decision because I don't know what careers will look like in the future.” Participants were asked to rate the degree to which the difficulty represented by each item described them on a 9-point scale from 1 (does not describe me) to 9 (describe me well), with higher scores indicating more difficulties experienced. A recent study by Leung et al. (2021) reported Cronbach's alpha of 0.95 for the full scale, and 0.74, 0.96, and 0.92 for the Readiness, Lack of Information, and Inconsistent Information subscales, respectively, in a sample of Hong Kong secondary students. In addition to the CDDQ, we also used an item from Gati et al. (2001) that was used to evaluate the effectiveness of a CACGS. The item asked respondents to indicate the status of their career decision after a CACGS intervention. Five options representing increasing level of decision clarity were presented, which were: (a) “I do not even have a general direction;” (b) “I have only a general direction;” (c) “I am deliberating on a number of occupations;” (d) “I am considering an occupation, but I would like to receive information about additional occupations;” and (e) “I am interested in a certain occupation, but I would like to receive more information about it.” A score of 1–5 was generated from the response with higher score indicating higher degree of decision clarity.

#### Education Aspirations

An item was developed to measure educational aspirations. Participants were asked “within one year after completing secondary school, will you consider the following options?” There was a total of 12 options (government supported universities, private universities, associate degree programs, higher diploma programs, vocational colleges, vocational diploma program, youth pre-employment training program, secondary diploma-equivalence study program, post-secondary education in mainland, post-secondary education in Taiwan, post-secondary education overseas, and full-time work). Respondents could indicate their intention to consider any of the options using one of the three choices: “will consider,” “will consider but have some hesitations,” or “will not consider.” The respondent could also indicate “do not know” and the response was not coded (i.e., treated as no response). We collected views from teachers of the five pilot schools in formulating the 12 options of this item to ensure that they represented the full spectrum of further education and career options that students would aspire upon completing secondary schools. Items in the exit survey including this item was piloted with students and teacher to ensure clarity and relevance. Two different education aspiration scores were derived from this set of items. The first (referred to as “intention to pursue government supported universities” score) was whether the respondent would consider “local universities supported by the government.” In Hong Kong, entrance to government supported universities was usually deemed most desirable due to financial and prestige considerations. The resultant score ranged from 1 to 3 with lower score indicating stronger intention to pursue. The second education aspiration score (referred to as “intention to pursue all university options” score) was obtained by aggregating respondents' intention to pursue all university options, including local-government supported, local-private, mainland, Taiwan, and other overseas options (total of five options). The resultant score ranged from 5 to 15 (five options each with three possible responses), with lower scores indicating stronger intention to consider the different options.

#### Understanding of Career Planning

Five options were presented on a scale of 1–5, with the following anchors: “I know nothing about what is career planning,” “I know a little bit about what is career planning,” “I have basic understanding on what is career planning,” “I know what is career planning even though I still have some questions,” and “I know quite well what is career planning and I could implement it on my life.” The resultant score ranged from 1 to 5 with higher score indicating perceptions of better understanding. Career planning is an adjunct learning area in secondary schools and this item aimed to capture the perception from students on their understanding. We piloted the items of the exit survey with students and teachers and they felt that the description, anchors, and the options were clear.

### Statistical Methods

Multivariate analysis of variance (MANOVA) was used to examine H1 and H2a. Infinity user status (users and non-users), gender (male and female), and school banding (from 1 to 3) were treated as independent variables, and the CAAS (H1) and CDDQ subscales (H2a) were treated as dependent variables. Univariate analysis of variance (ANOVA) was used to examine the hypotheses related to decision clarity (H2b), aspiration to pursue university education (H3a), and understanding of career planning (H3b), using the same set of independent variables as in the MANOVA analyses. Significant user status main effects and non-significant interaction effects (involving user status) would support the hypotheses (i.e., no variation of effects across gender and school banding). The SPSS statistical software (version 27) was used to run the analyses. Effect size was measured by the *partial eta squared*. We used Cohen's (1988) suggestions as a general “rule-of-thumb” to interpret effect size in relation to *partial eta squared*, with value of 0.002 or lower being considered as low effect, value of 0.006 as medium effect, and value of 0.014 as strong effect.

## Results

**Figure 2:**
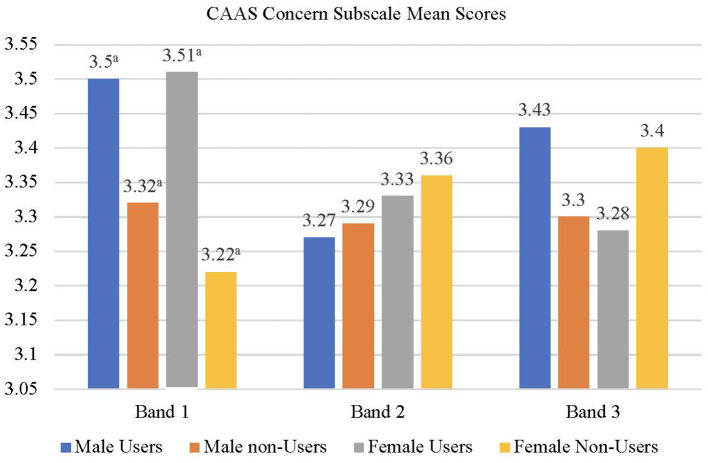
User status × school banding interaction of the CAAS—concern subscale. *N* = 2,935 12th graders (1,549 females and 1,374 males, 12 unidentified; 844 users and 2,091 non-users). CAAS, Career Adaptability Scale. Scores ranged from 1 to 5 with higher scores indicating higher level of adaptability resources. ^a^Users from band 1 schools had significantly higher scores than non-users from the same banding (*p* < 0.001).

**Figure 3:**
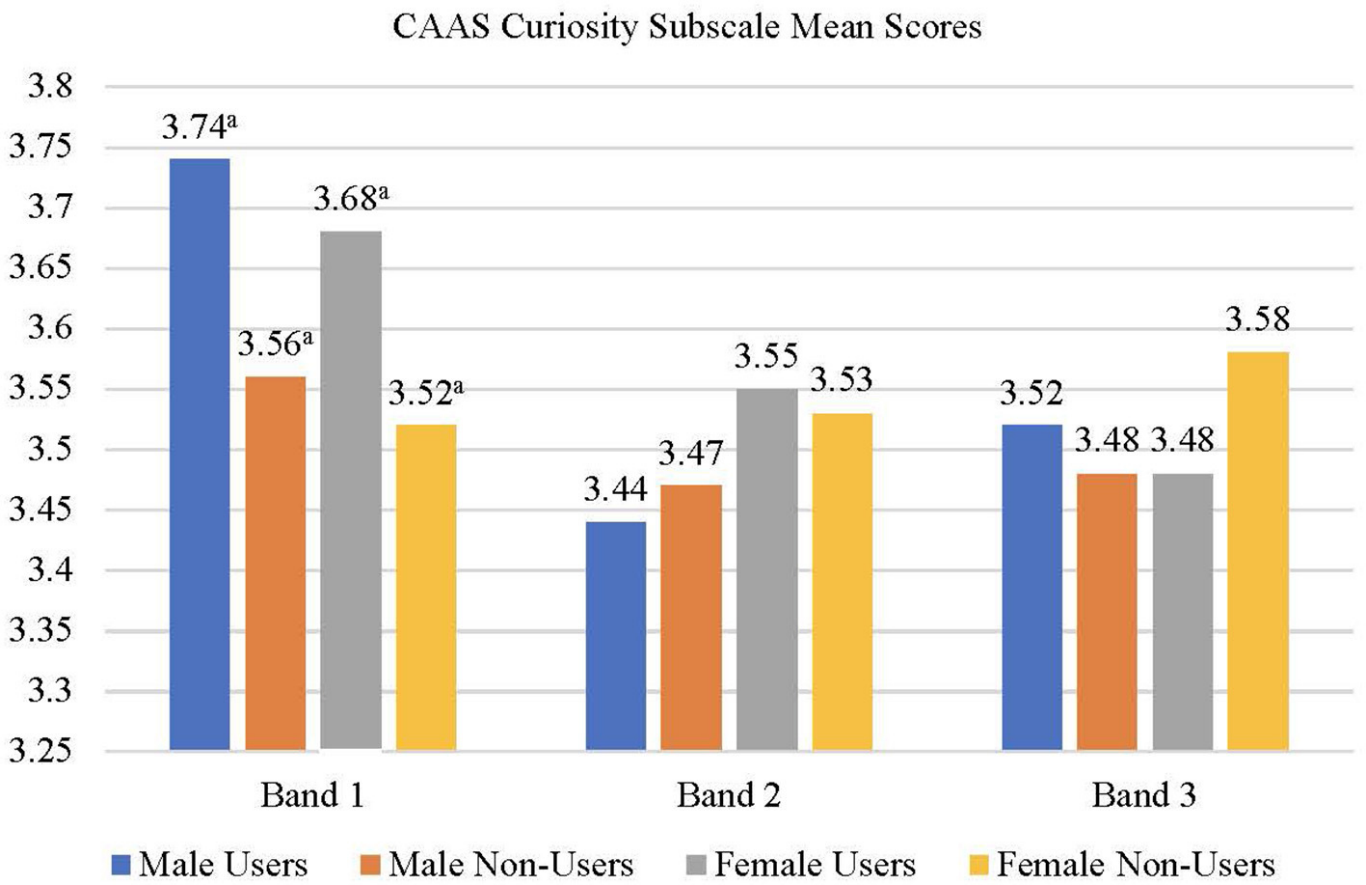
User status × school banding interaction of the CAAS—curiosity subscale. *N* = 2,935 12th graders (1,549 females and 1,374 males, 12 unidentified; 844 users and 2,091 non-users). CAAS, Career Adaptability Scale. Scores ranged from 1 to 5 with higher scores indicating higher level of adaptability resources. ^a^Users from band 1 schools had significantly higher scores than non-users from the same banding (*p* < 0.01).

**Figure 4:**
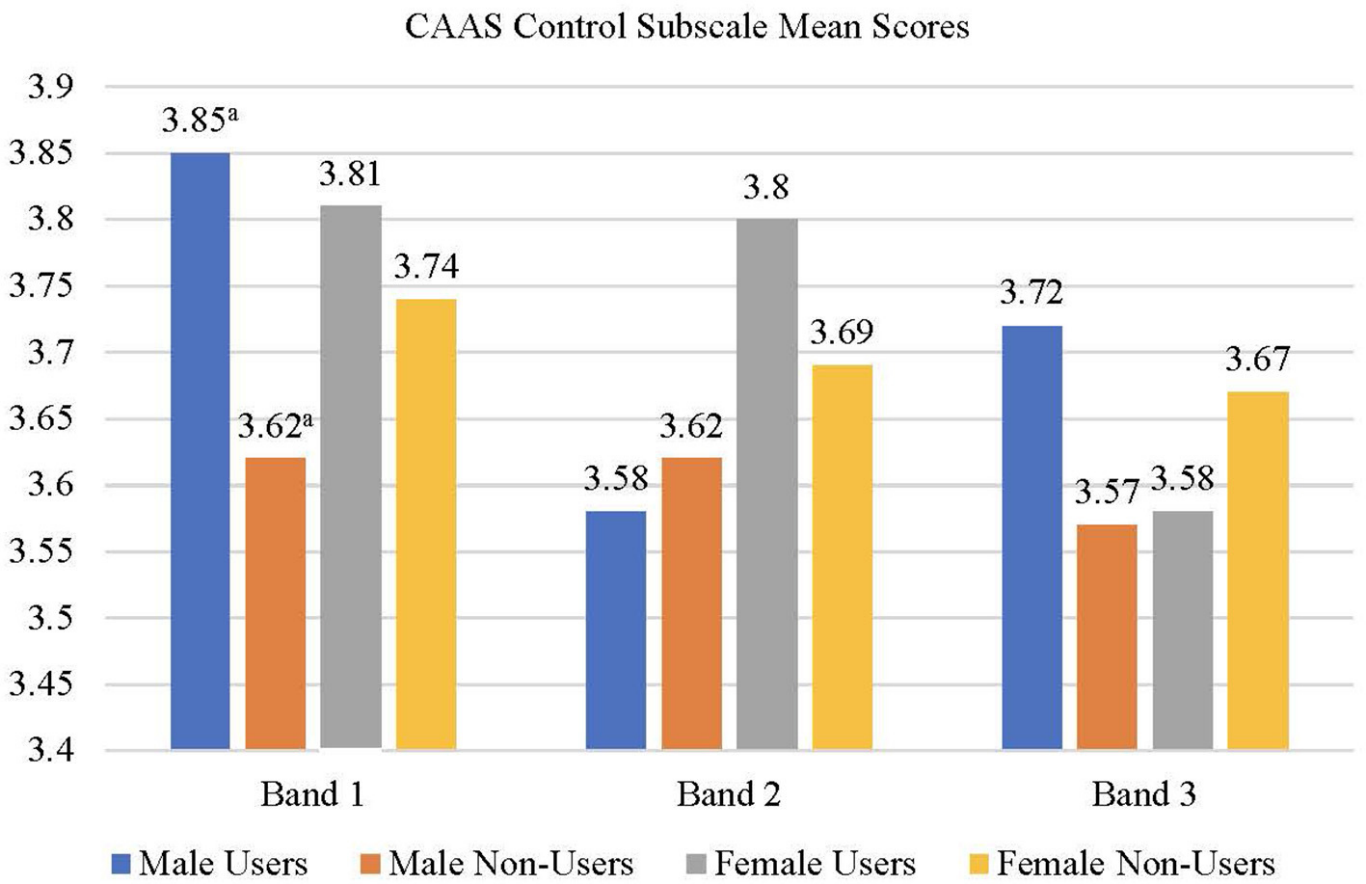
User status × gender × school banding interaction of the CAAS—control subscale. *N* = 2,935 12th graders (1,549 females and 1,374 males, 12 unidentified; 844 users and 2,091 non-users). CAAS, Career Adaptability Scale. Scores ranged from 1 to 5 with higher scores indicating higher level of adaptability resources. ^a^Male users from band 1 schools had significantly higher scores than male non-users from the same school banding (*p* < 0.05).

The correlation among the measures as well as the means and standard deviations of scores are presented in Tables 4, 5, respectively. A user status × gender × school banding MANOVA was conducted with the CAAS subscale scores (concern, control, curiosity, and confidence) as dependent variables. None of the main effects were significant but the user status × school banding [*F*_(4,2,908)_ = 2.08, *p* < 0.05, *Wilks' Lambda* = 0.994, *partial eta squared* = 0.003] and user status × gender × school banding interactions [*F*_(3,2,909)_ = 1.92, *p* = 0.05, *Wilks' Lambda* = 0.995, *partial eta squared* = 0.003] were significant suggesting that the effects of user status on the dependent variables varied across gender and school banding groups. Univariate follow-up analyses suggested that the user status × school banding interaction was significant for the concern (*p* < 0.01) and curiosity (*p* < 0.05) subscales of the CAAS. For the concern (Figure 2) and curiosity (Figure 3) subscales, male and female users from band 1 schools had significantly higher scores than non-users from the same school banding (*p* < 0.01), yet the difference was not significant for students in other school bandings. Univariate follow-up analysis on the user status × gender × banding interaction revealed that the interaction was significant only for the control subscale (*p* < 0.05) (Figure 4). For this subscale, further comparisons suggested that there were significant differences between male users and non-users in band 1 schools only (*p* < 0.05). Overall, findings suggested that the helpfulness of Infinity varied across school banding, and H1 was only partially.

**Table 4:** Intercorrelations among measures.

	**1**	**2**	**3**	**4**	**5**	**6**	**7**	**8**	**9**	**10**	**11**	**12**	**13**
1.CAAS—Total	-	0.82^**^	0.86^**^	0.87^**^	0.86^**^	−0.17^**^	0.00	−0.22^**^	−0.17^**^	0.33^**^	−0.13^**^	−0.06^**^	0.35^**^
2.Concern		-	0.61^**^	0.62^**^	0.58^**^	−0.18^**^	−0.01	−0.25^**^	−0.16^**^	0.32^**^	−0.09^**^	−0.06^**^	0.34^**^
3.Control			-	0.66^**^	0.65^**^	−0.16^**^	−0.03	−0.18^**^	−0.16^**^	0.28^**^	−0.12^**^	−0.03	0.26^**^
4.Curiosity				-	0.70^**^	−0.11^**^	−0.03	−0.16^**^	−0.11^**^	0.27^**^	−0.10^**^	−0.05^**^	0.30^**^
5.Confidence					-	−0.14^**^	−0.02	−0.19^**^	−0.14^**^	0.26^**^	−0.12^**^	−0.06^**^	0.29
6.CDDQ - Total						-	0.75^**^	0.94^**^	0.92^**^	−0.38^**^	0.05^**^	−0.04	−0.27^**^
7.Readiness							-	0.58^**^	0.57^**^	−0.18^**^	0.04	−0.05^**^	−0.12^**^
8.Lack-Info								-	0.81^**^	−0.41^**^	0.04	−0.05^**^	−0.30^**^
9.Inconsist									-	−0.35^**^	0.06^**^	−0.05^**^	−0.24^**^
10.DStatus										-	−0.12^**^	−0.05^**^	0.46^**^
11.Intent-Local											-	0.55^**^	−0.12^**^
12.Intent-All												-	−0.08^**^
13.Understand													-

*N = 2,935 12th graders (1,549 females and 1,374 males, 12 unidentified; 844 users and 2,091 non-users)*.

*CAAS Total, Career Adaptability Scale total score. Concern, Control, Curiosity, and Confidence are subscales of CAAS; CDDQ Total, Total score of the Career Decision-Making Difficulties Questionnaire; Readiness, Readiness subscale of CDDQ; Lack-Info, Lack of Information subscale of CDDQ; Inconsist, Inconsistent subscale of CDDQ; DStatus, Career decision status; Intent-local, Intention to pursue government supported universities; Intent-All, Intention to pursue all university options; Understand, Understanding of career planning*.

** *p < 0.01*.

**Table 5:** Means and standard deviations of career development measures of participants who were users of infinity and non-users.

	**Male**		**Female**	
**Measures**	**Users**	**Non-users**	**Users**	**Non-users**
	**Mean (SD)**	**Mean (SD)**	**Mean (SD)**	**Mean (SD)**
1.CAAS—Total	3.58 (0.62)	3.49 (0.62)	3.58 (0.57)	3.55 (0.56)
Band 1 schools	3.72 (0.54)	3.54 (0.60)	3.69 (0.56)	3.52 (0.60)
Band 2 schools	3.45 (0.62)	3.49 (0.58)	3.58 (0.55)	3.55 (0.55)
Band 3 schools	3.59 (0.67)	3.49 (0.68)	3.48 (0.58)	3.56 (0.57)
2.Concern^e^	3.39 (0.73)	3.30 (0.73)	3.37 (0.70)	3.35 (0.70)
Band 1 schools	3.50 (0.71)	3.32 (0.78)	3.51 (0.76)	3.22 (0.78)
Band 2 schools	3.27 (0.70)	3.29 (0.70)	3.33 (0.68)	3.36 (0.68)
Band 3 schools	3.43 (0.79)	3.30 (0.73)	3.28 (0.64)	3.40 (0.69)
3.Control^d^	3.72 (0.75)	3.60 (0.74)	3.61 (0.71)	3.69 (0.72)
Band 1 schools	3.85 (0.71)	3.62 (0.71)	3.81 (0.72)	3.74 (0.80)
Band 2 schools	3.58 (0.72)	3.62 (0.71)	3.80 (0.75)	3.69 (0.71)
Band 3 schools	3.72 (0.78)	3.57 (0.80)	3.58 (0.73)	3.67 (0.70)
4.Curiosity^e^	3.56 (0.71)	3.49 (0.71)	3.60 (0.70)	3.54 (0.63)
Band 1 schools	3.74 (0.63)	3.56 (0.68)	3.68 (0.71)	3.52 (0.71)
Band 2 schools	3.44 (0.71)	3.47 (0.68)	3.55 (0.65)	3.53 (0.60)
Band 3 schools	3.52 (0.76)	3.48 (0.75)	3.48 (0.71)	3.58 (0.64)
5.Confidence	3.66 (0.71)	3.60 (0.71)	3.67 (0.67)	3.60 (0.67)
Band 1 schools	3.77 (0.62)	3.65 (0.66)	3.77 (0.60)	3.60 (0.71)
Band 2 schools	3.55 (0.78)	3.59 (0.68)	3.64 (0.69)	3.61 (0.67)
Band 3 schools	3.68 (0.72)	3.57 (0.79)	3.57 (0.71)	3.59 (0.64)
6.CDDQ—Total	4.48 (1.26)	4.66 (1.15)	4.44 (1.19)	4.59 (1.16)
Band 1 schools	4.32 (1.28)	4.55 (1.24)	4.25 (1.13)	4.59 (1.24)
Band 2 schools	4.67 (1.07)	4.67 (1.12)	4.39 (1.25)	4.56 (1.13)
Band 3 schools	4.44 (1.44)	4.68 (1.18)	4.66 (1.17)	4.64 (1.17)
7.Readiness^a, b, c^	4.67 (1.03)	4.81 (0.94)	4.55 (0.92)	4.69 (0.91)
Band 1 schools	4.58 (0.94)	4.80 (0.97)	4.48 (0.88)	4.71 (1.00)
Band 2 schools	4.85 (0.92)	4.82 (0.91)	4.44 (0.97)	4.67 (0.89)
Band 3 schools	4.53 (1.23)	4.81 (0.97)	4.71 (0.90)	4.73 (0.90)
8.Lack-Info^a, b, c^	4.52 (1.64)	4.70 (1.51)	4.47 (1.60)	4.68 (1.55)
Band 1 schools	4.26 (1.70)	4.58 (1.59)	4.19 (1.54)	4.62 (1.72)
Band 2 schools	4.76 (1.45)	4.73 (1.45)	4.50 (1.57)	4.66 (1.50)
Band 3 schools	4.50 (1.76)	4.72 (1.56)	4.73 (1.55)	4.73 (1.57)
9.Inconsist^a, b, c^	4.25 (1.54)	4.44 (1.40)	4.28 (1.44)	4.37 (1.42)
Band 1 schools	4,13 (1.62)	4.28 (1.53)	4.11 (1.41)	4.42 (1.48)
Band 2 schools	4.37 (1.37)	4.44 (1.34)	4.20 (1.48)	4.44 (1.34)
Band 3 schools	4.26 (1.66)	4.50 (1.44)	4.53 (1.42)	4.45 (1.44)
10.DStatus^a, b, c^	3.62 (1.20)	3.36 (1.22)	3.72 (1.08)	3.62 (1.15)
Band 1 schools	3.73 (1.21)	3.54 (1.18)	3.95 (1.02)	3.75 (0.98)
Band 2 schools	3.51 (1.21)	3.39 (1.20)	3.74 (1.08)	3.63 (1.18)
Band 3 schools	3.64 (1.17)	3.25 (1.25)	3.49 (1.09)	3.55 (1.17)
11.Intent-Local^a, b, c, e^	1.32 (0.61)	1.46 (0.72)	1.25 (0.58)	1.34 (0.65)
Band 1 schools	1.12 (0.43)	1.07 (0.33)	1.08 (0.37)	1.07 (0.31)
Band 2 schools	1.33 (0.58)	1.39 (0.67)	1.24 (0.55)	1.31 (0.64)
Band 3 schools	1.54 (0.75)	1.74 (0.80)	1.42 (0.71)	1.53 (0.74)
12.Intent-All	9.93 (2.24)	10.34 (2.23)	9.96 (1.97)	9.97 (2.08)
Band 1 schools	9.92 (2.02)	9.74 (1.93)	9.87 (1.68)	9.68 (1.81)
Band 2 schools	9.92 (1.96)	10.25 (2.12)	9.96 (2.00)	10.0 (2.02)
Band 3 schools	9.95 (2.77)	10.71 (2.43)	10.06 (2.21)	10.0 (2.33)
13.Understand^a, b, c^	3.36 (0.84)	3.19 (0.87)	3.47 (0.82)	3.33 (0.80)
Band 1 schools	3.42 (0.88)	3.29 (0.85)	3.63 (0.82)	3.42 (0.80)
Band 2 schools	3.28 (0.80)	3.23 (0.83)	3.42 (0.81)	3.29 (0.79)
Band 3 schools	3.39 (0.86)	3.11 (0.94)	3.36 (0.80)	3.37 (0.82)

*N = 2,935 12th graders (1,549 females and 1,374 males, 12 unidentified; 844 users and 2,091 non-users)*.

*CAAS Total, Career Adaptability Scale total score. Concern, Control, Curiosity, and Confidence are subscales of CAAS; CDDQ Total, Total score of the Career Decision-Making Difficulties Questionnaire; Readiness, Readiness subscale of CDDQ; Lack-Info, Lack of Information subscale of CDDQ; Inconsist, Inconsistent subscale of CDDQ; DStatus, Career decision status; Intent-local, Intention to pursue government supported universities; Intent-All, Intention to pursue all university options; Understand, Understanding of career planning*.

*#1 to #5, #10, and #13 – Higher scores indicate higher ratings on the designated variables*.

*#6 to #9 – Higher scores indicate more decision-making difficulties*.

*#11 and #12 – Lower scores indicate stronger intention (#11 scores ranged from 1 to 3, #12 scores ranged from 5 to 15)*.

a *User status main effect*.

b *Gender main effect*.

c *School banding main effect*.

d *User status × gender × school banding interaction effect*.

e *User status × school banding interaction effect*.

Similar to the analysis on the CAAS scores, a user status × gender × school banding MANOVA was conducted with the CDDQ subscale scores as the dependent variables (readiness, lack of information, and inconsistent information). There was a user status main effect [*F*_(3,2,909)_ = 4.89, *p* = 0.01, *Wilks' Lambda* = 0.995, *partial eta squared* = 0.005]. Univariate follow-up analyses with each of the CDDQ subscales as dependent variables suggested that there were significant differences between the two user status groups in all the three subscales (*p* < 0.01). Overall, users of the Infinity system reported lesser career decision-making difficulties than those who did not use the system. The gender [*F*_(3,2,909)_ = 3.64, *p* < 0.05, *Wilks' Lambda* = 0.996, *partial eta squared* = 0.004] and school banding [*F*_(3,2,909)_ = 2.82, *p* = 0.01, *Wilks' Lambda* = 0.994, *partial eta squared* = 0.003] main effects were both significant, as well as the gender × banding interaction [*F*_(3,2,909)_ = 2.18, *p* = 0.05, *Wilks' Lambda* = 0.996, *partial eta squared* = 0.002]. Male students in band 2 schools had the highest CDDQ scores but for female students, students in band 3 schools expressed the highest difficulties (Table 5). Yet none of the interactions involving user status was significant indicating that the effects of using the Infinity system did not varied across gender and school banding. Hypothesis 2a was supported.

We run a user status × gender × school banding ANOVA to compare the career decision-making status of students using the decision clarity item from Gati et al. (2001) as the dependent variable. Students who used the Infinity system reported a higher degree of decision clarity than students who did not used the system [*F*_(1,2,918)_ = 9.39, *p* > 0.01, *partial eta squared* = 0.003]. Male students expressed greater decision clarity than female students [*F*_(1,2,918)_ = 11.43, *p* > 0.001, *partial eta squared* = 0.004]. There was also a school banding main effect [*F*_(1,2,918)_ = 7.87, *p* > 0.001, *partial eta squared* = 0.005]. Multiple comparisons revealed that students from band 1 schools expressed stronger decision clarity than other students. However, none of the interaction effects were significant. Findings supported hypothesis 2b.

**Figure 5:**
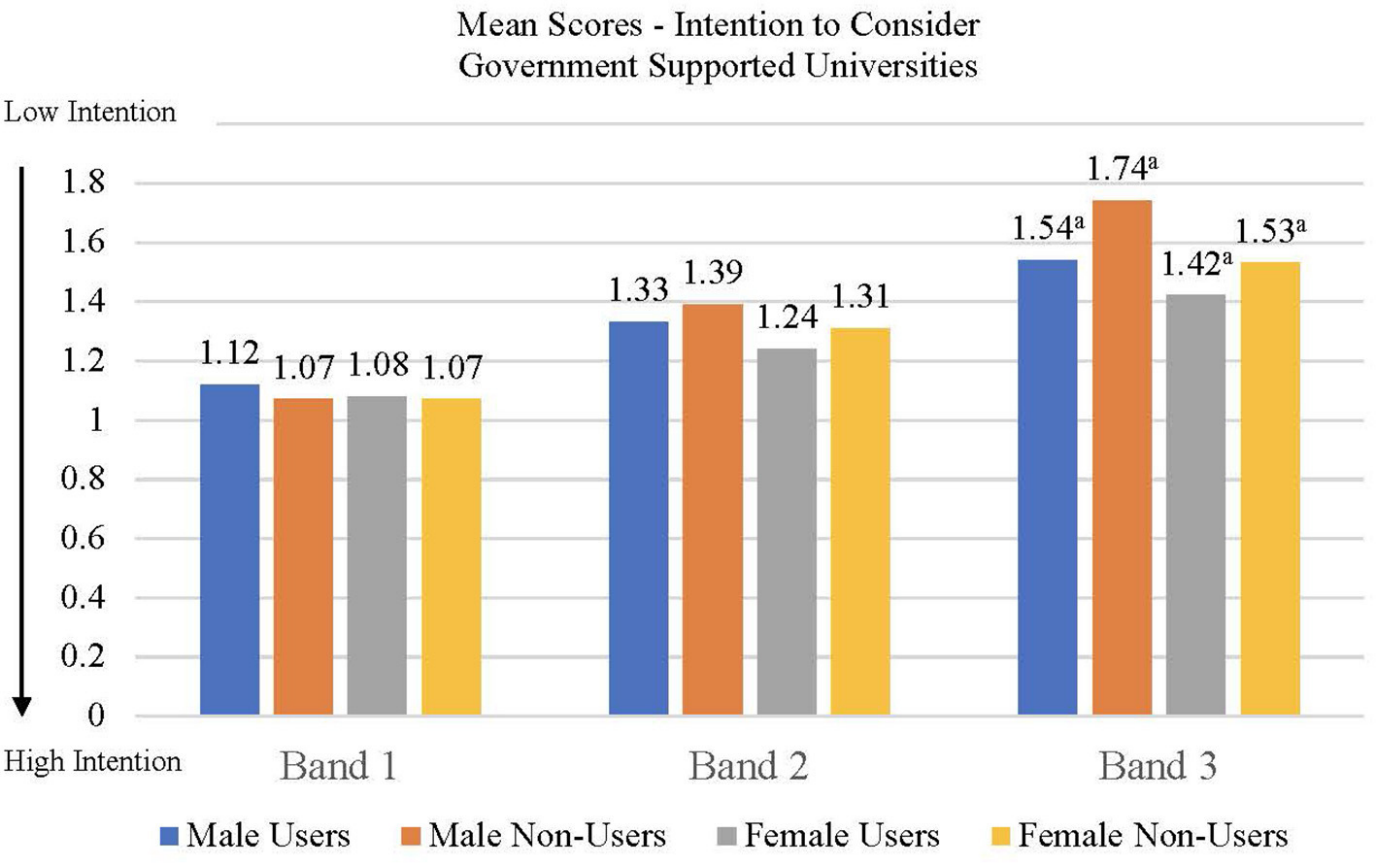
User status × school banding interaction of the intention to consider government supported universities item. *N* = 2,935 12th graders (1,549 females and 1,374 males, 12 unidentified; 844 users and 2,091 non-users). Scores ranged from 1 to 3 with *lower scores indicating higher intention* to consider government supported universities. ^a^Users from band 3 schools had significantly lower scores (i.e., higher intention) than non-users from the same school banding (*p* < 0.01).

A user status × gender × school banding ANOVA was done with aspiration to attend government supported universities as the dependent variable. The user status main effect was significant [*F*_(1,2,983)_ = 4.75, *p* < 0.05, *partial eta squared* = 0.002] but the user status x school banding interaction effect was also significant [*F*_(1,2,983)_ = 3.30, *p* < 0.05, *partial eta squared* = 0.002]. Users from band 3 schools expressed greater intention to pursue government supported universities than non-users from the same school banding (*p* < 0.01) (Figure 5), yet there was no difference between users and non-users among students from other school bandings. There was a significant gender main effect [*F*_(1,2,983)_ = 9.51, *p* < 0.01, *partial eta squared* = 0.003] and female students express stronger intention than male students. The school banding main effect was also significant [*F*_(1,2,983)_ = 82.6, *p* < 0.001, *partial eta squared* = 0.054], and multiple comparisons suggested that students from higher banding schools expressed stronger intention. The analyses on all the university options combined suggested that there was only a school banding main effect [*F*_(1,2,712)_ = 4.60, *p* < 0.01, *partial eta squared* = 0.003] with students from band 1 schools expressed greater consideration of these options than students from schools. The user status main effect was not significant. Overall, findings partially supported hypothesis H3a.

We asked students to indicate on a scale of 1–5 on their understanding of what was career planning. A user status × gender × school banding ANOVA was done with this item as the dependent variable. Students who used the Infinity system perceived better understanding of career planning than those who did not use the system [*F*_(1,2,898)_ = 12.71, *p* < 0.001, *partial eta-squared* = 0.004]. There were gender differences and female students perceived better understanding than male students [*F*_(1,2,898)_ = 12.16, *p* < 0.001, *partial eta squared* = 0.004]. The school banding main effect was also significant [*F*_(1,2,898)_ = 5.25, *p* < 0.01, *partial eta-squared* = 0.004]. Follow-up analysis revealed that students from band 1 schools expressed a greater understanding than students from other schools. However, none of the interaction effects were significant. Findings supported hypothesis H3b.

## Discussion

Findings show that the Infinity system was useful and helpful to students but there were variations across academic background of schools and gender. Several themes emerged. First, differences in career adaptability between users and non-users were observed from students studying in band 1 schools (concern and curiosity subscales, control subscale for male students only) but not from students studying in band 2 and band 3 schools. Adaptability resources are viewed as competencies in the adaptability process (Savickas, 2020) and students in band 1 schools might benefit from using the Infinity system because of their stronger academic competencies compared to other students. Second, Infinity might have strengthened the intentions of students from band 3 schools to pursue government supported universities. The process of career- and self-exploration should encourage some students to aspire for challenging educational opportunities. Post-secondary education is an important step that could open up career opportunities and pathways for students, especially for students in band 3 schools who were likely to come from economically disadvantaged backgrounds. Third, students in band 2 schools might have benefited the least compared to students in other school bandings. Band 2 tended to experience a high level of academic pressure as they might not be as confident as students in band 1 schools in reaching their higher-education goals. At the same time, they might not be as open to alternative post-secondary pathways like their counterparts in band 3 schools. Accordingly, students in band 2 schools might have needs that required a deeper level of support than those provided through the Infinity platform. Fourth, the Infinity system was found to be helpful to female and male students. The only gender difference was on the control subscale of the CAAS, where differences between users and non-users were found only among male students in band 1 schools. The identified differences underscore the importance of understanding the needs of different groups of students, including male and female students and students from different academic backgrounds. Fifth, findings on career decision-making and decision clarity were rather consistent across gender and school banding and the effects were uniform across participants. Last, even though the effect sizes of statistical differences were low to medium (Cohen, 1988), all the findings were consistently in the same direction.

The positive outcomes observed are likely to be connected to both the quantitative and qualitative functions of Infinity which are designed as building blocks to facilitate self-awareness and career-meaning construction. For instance, the career assessment and dashboard function of Infinity should contribute to students' readiness as well as their self- and career-knowledge. The qualitative functions (e.g., career curriculum) facilitated the integration and alignment of diverse information instrumental to decision clarity (McMahon et al., 2020). The need analysis that preceded the development of Infinity (Ho and Leung, 2016; Leung, 2020a) showed that tools and resources were greatly needed for career guidance in Hong Kong. The availability of new tools from Infinity played a role in empowering students and building up their sense of concern and control toward the unknown future.

### Practice Implications

Even though the Infinity system was developed with senior secondary students in mind, the lessons learned and the implications derived from the experience could be applied to future CACGS developed for other target groups or organizational settings.

First, Infinity shows that it is possible to create a CACGS using a constructivist framework. Career assessment is used as ingredients for meaning and self-identity construction. Infinity offers users with multiple tools to deepen their explorations and understanding (McMahon et al., 2020), and to integrate their insights from assessment and other sources of observations into life stories and action plans (see examples in Table 3). A CACGS with only the assessment and information components would not be able to achieve these objectives.

Second, Infinity is an example of a self-directed career planning system where users could use and reuse the various applications to work out their next career plans, or to take adaptive actions to meet new career challenges (Lent and Brown, 2013). In a fast changing and unpredictable world, self-directedness is required and career planning has to be a continuous process of adapting and congruence-fitting. Infinity offers users a platform where they could prepare for their transitions without intensive support from teachers and counselors. They could review and update the cumulative information already assembled in the system.

Third, the system can be used to serve users with diverse preferences and needs. Users could use the media modalities they prefer to express and articulate their career stories. For instance, students with special educational needs might not prefer tools and functions with extensive verbal or written content. They could use images and sounds to communicate, express, and record their career stories (e.g., the “My Gallery” function, Table 1). The gamified process also goes beyond the verbal and cognitive channels of operation and encourage users to be more fully engaged (e.g., visual, physical coordination). Through these tools, the Infinity system opens up multiple avenues of qualitative exploration to address the needs and preferences of diverse users.

Fourth, even though Infinity could be used independently without professional support, it is a versatile system that could be complement existing career development services (Vigurs et al., 2017). Users and counselors could work collaboratively in the career development process using Infinity as a supportive platform. In a school setting, career teachers could use the tools in Infinity (e.g., interest assessment, “Shine ∙ My Profile,” the Career Adventure Report; see Table 1) to guide continuous individual career. The career education curriculum (Table 3) allows teachers and students to make use of all the functions of Infinity to engage in the steps of career planning. Cross-curricular activities (e.g., workplace learning connected to STEM classes, writing an essay about work values for language classes) could be developed, recorded, and synthesized through the Infinity platform. These cross-curricular activities could enlighten students on how school subjects are connected to diverse careers. In an organizational setting, a similar CACGS could help users and career coaches to work collaboratively in career development initiatives, to record insights from professional development activities, and to use their preferred multi-media format to express their career development themes.

Meanwhile, schools and organizations could use the aggregate information from Infinity to strengthen career services. For instance, the aggregate career development data from Infinity system about a cohort of students could inform schools and career teachers about the needs of students and the focus of future career programs. Students with special career development needs could be identified and on-target interventions could be offered (e.g., special guidance for students with an interest-competence mis-match). The aggregate data could be used to measure and identify progress (e.g., whether students have engaged in meaningful workplace encounters), or to meet specific career guidance benchmarks (e.g., Gatsby Charitable Foundation, 2018).

### Future Possibilities

There are many ways that a system such as Infinity could be further enhanced. On the conceptual side, the constructivist framework could be more deeply ingrained into the system. For instance, users could create career stories with different levels of sophistication and those with more experience with the tools could work on mini-stories around the main plot. Layers of short and focused learning modules with adaptive actions could be embedded into the career stories construction process to allow users (e.g., students with different academic backgrounds) to progressively learn career planning skills that are core to the constructivist perspectives (e.g., reflexivity, narratability, adaptability, and intentionality). In short, through using our knowledge in learning sciences and curriculum development (Kalantzis and Cope, 2012), it is possible to create a series of interlocking learning and action episodes that could maximize the beneficial effects of CACGS.

On the technical side, Infinity could be further informed by advances in educational measurement and ICT technologies. New measurement models such as adaptive assessment and item-response theory could substantially improve the precision of measures (Tracey, 2020). Machine learning and analytics derived from big-data (i.e., based on the accumulated data from Infinity) could unveil patterns of career development and user characteristics that are connected to different outcomes. These new discoveries could inform users and service providers on how career education materials could best be designed in a digital system, and what interventions are optimal for users with different needs (Kalantzis and Cope, 2012). The career development search engine could be strengthened. Tailored and personalized information from the latest (e.g., based on one's unique interests and preferences) could be sent to users to keep them engaged and informed. Meanwhile, user journey inquiries could be conducted to understand how the gamified learning process could be optimized to make the process of using the CACGS more engaging, fun and user-friendly.

There are plenty of possibilities for research to examine the effectiveness of CACGS similar to the Infinity system. First, research could examine how school-based career guidance provisions supported by fit-for-purpose technology (e.g., the Infinity system) could boost and sustain positive effects to students with different academic backgrounds (Vigurs et al., 2017). Second, research needs to understand how counselor training using a constructivist conceptual model (e.g., life-design career counseling of CCT) could facilitate the beneficial use of CACGS (Kettunen and Sampson, 2019). Third, Future research could compare the effects of using the career assessment functions of CACGS with those using the full-spectrum of applications informed by the constructivist framework, and to identify students who might benefit the most from these two modes of interventions. Fourth, qualitative research could be done to complement quantitative findings to understand user experiences and the specific ways that the digital system was helpful to different users (students from different schools and backgrounds).

This study offers initial findings on the positive effects of using the Infinity system with senior secondary students. The results are the first step toward using more rigorous experimental designs to test the effects of using technology system to support career development. These experimental approaches should include random assignments of participants to experimental and control groups, and collection of pre- and post-intervention data. In this study, there were variations among students on their usage of the applications in the Infinity system (i.e., users did not use all the applications or the same set of applications) and the effects of singular application were not assessed. Future studies should evaluate the key applications of the system to understand the unique and specific effects to users. Learning analytics could be collected to identify learning pathways and the key factors (e.g., user characteristics, curriculum designs) that contributed to positive outcomes of student from different backgrounds (e.g., school banding). These additional studies should help to advance the scientific foundation of CACGS and address the limitations of the present study.

## Summary and Conclusion

The Infinity is an innovative CACGS based on a constructivist framework (i.e., career construction theory). It is a platform created for meaning construction in a digital-age where individuals could learn, plan, play, and manage their career development simultaneously and continuously. The quantitative and qualitative tools of Infinity aim to strengthen awareness, competencies, and actions to cope with career transitions. Users could use the Infinity with or without professional support. They could connect with peers, teachers, and parents for empowerment. Initial findings support the positive effects of using the Infinity system.

## Author's Note

Part of this article was done while the author serves as Visiting Professor, Center for Asian American Studies, College of Education, University of Houston.

## Data Availability Statement

The raw data supporting the conclusions of this article will be made available by the authors, without undue reservation.

## Ethics Statement

The studies involving human participants were reviewed and approved by Survey Research Ethics Committee, CUHK. Written informed consent to participate in this study was provided by the participants' legal guardian/next of kin.

## Author Contributions

The author confirms being the sole contributor of this work and has approved it for publication.

## Funding

This research was supported by the Hong Kong Jockey Club Charities Trust as part of the Career and Life Adventure Planning Project (CLAP for Youth @ JC).

## Conflict of Interest

The author declares that the research was conducted in the absence of any commercial or financial relationships that could be construed as a potential conflict of interest.

## Publisher's Note

All claims expressed in this article are solely those of the authors and do not necessarily represent those of their affiliated organizations, or those of the publisher, the editors and the reviewers. Any product that may be evaluated in this article, or claim that may be made by its manufacturer, is not guaranteed or endorsed by the publisher.
